# Urinary arsenic profiles reveal exposures to inorganic arsenic from private drinking water supplies in Cornwall, UK

**DOI:** 10.1038/srep25656

**Published:** 2016-05-09

**Authors:** D. R. S. Middleton, M. J. Watts, E. M. Hamilton, E. L. Ander, R. M. Close, K. S. Exley, H. Crabbe, G. S. Leonardi, T. Fletcher, D. A. Polya

**Affiliations:** 1 School of Earth, Atmospheric and Environmental Sciences & Williamson Research Centre for Molecular Environmental Science, University of Manchester, Oxford Rd, Manchester, M13 9PL, UK; 2 Inorganic Geochemistry, Centre for Environmental Geochemistry, British Geological Survey, Nicker Hill, Keyworth, Nottinghamshire, NG12 5GG, UK; 3 Centre for Radiation, Chemicals and Environmental Hazards (CRCE), Public Health England, Chilton, Didcot, Oxfordshire, OX11 0RQ, UK

## Abstract

Private water supplies (PWS) in Cornwall, South West England exceeded the current WHO guidance value and UK prescribed concentration or value (PCV) for arsenic of 10 μg/L in 5% of properties surveyed (*n* = 497). In this follow-up study, the first of its kind in the UK, volunteers (*n* = 207) from 127 households who used their PWS for drinking, provided urine and drinking water samples for total As determination by inductively coupled plasma mass spectrometry (ICP-MS) and urinary As speciation by high performance liquid chromatography ICP-MS (HPLC-ICP-MS). Arsenic concentrations exceeding 10 μg/L were found in the PWS of 10% of the volunteers. Unadjusted total urinary As concentrations were poorly correlated (Spearman’s ρ = 0.36 (P < 0.001)) with PWS As largely due to the use of spot urine samples and the dominance of arsenobetaine (AB) from seafood sources. However, the osmolality adjusted sum, U-As^IMM^, of urinary inorganic As species, arsenite (As^III^) and arsenate (As^V^), and their metabolites, methylarsonate (MA) and dimethylarsinate (DMA), was found to strongly correlate (Spearman’s ρ: 0.62 (P < 0.001)) with PWS As, indicating private water supplies as the dominant source of inorganic As exposure in the study population of PWS users.

Chronic exposure to arsenic (As) in drinking water is a well-documented cause of numerous cancerous and non-cancerous health defects^1^, including cancers of the lung, bladder and skin. While most cases of chronic As exposure in drinking water have been reported in Bangladesh and West Bengal^2^,^3^, countries on all continents are affected^4^. Recent studies have identified lower (e.g.<150 As μg/L) exposures in European and North American populations in both municipal and private supplies in rural locations where centralized treated water supply has not been implemented. Examples of this scenario include Serbia^5^, Hungary^6^, Romania^7^, Slovakia^7^ and the USA^8^, where for the latter it is estimated that approximately 15% of the population rely on private groundwater supplies (PWS)^9^, and as much as 40% of people in New Hampshire, Vermont and Maine^10^. Many communities in rural parts of the UK also use PWS and a reported 567,261 people in the UK live or work in properties served by PWS^11^.

One area warranting further investigation is Cornwall in South West England, where PWS usage is estimated to range from 20,000–30,000 wells^12^ although only 2,462 single domestic PWS are currently registered on the Drinking Water Inspectorate (DWI) database^11^. Cornwall’s diverse geology and extensive history of mineral exploitation make it a region of elevated environmental inorganic As^13^, with an estimated 722 km^2^ of As contaminated land^14^. Elevated concentrations have previously been reported in soils^15^,^16^, stream waters^17^/sediments^18^ and household dusts^19^,^20^. In 2010, the Private Water Supplies Regulations (2009)^21^ came into force and prompted an initiative to investigate the possible public health implications of PWS consumption. The abovementioned findings and the high frequency of PWS in Cornwall relative to most of the UK led to its selection for a study^22^ investigating the trace metal content of UK PWS. This study found As in PWS drinking water exceeding the 10 As μg/L UK prescribed concentration or value^23^ (PCV) in 27 out of 497 (5%) households^22^. This suggests that a considerable number of people in the region may be subject to elevated levels of As in their drinking water, an exposure route not comprehensively investigated in Cornwall, nor indeed the UK as a whole, to date.

The identification of elevated concentrations of As in drinking water alone can help provide an indication of the population at risk. However, the use of exposure biomonitoring, the analysis of biological material for the presence of chemicals and their metabolites, allows for a more direct quantification of internal exposure^24^ underpinning environmental chemical attributable health risks. A common approach to As biomonitoring is the analysis of urine samples for inorganic arsenite (As^III^), arsenate (As^V^) and methylated metabolites methylarsonate (MA) and dimethylarsinate (DMA) that are excreted in the urine following metabolism in the liver^25^. It is accepted that post intake, inorganic As^V^ is reduced to As^III^ followed by methylation to MA and DMA^26^. The process was formerly considered to be a detoxification pathway, but findings of genotoxic intermediate trivalent forms of MA and DMA suggest otherwise^27^. The exact mechanisms of As biomethylation are subject to ongoing investigation^28^. For the purpose of exposure assessment, the methylation of inorganic to organic As species justifies the quantification of MA and DMA, whilst acknowledging that direct intake of both of these species from dietary sources has been reported^29^. The majority of As is excreted within 4 days of dosage^30^, making urinary As a useful measure of recent exposure, and has been used, for example, to demonstrate rice as a significant dietary exposure pathway in rice consumers in the UK^31^, USA^32^ and West Bengal^33^. Several studies have used urinary As to model the risk of health end-points and toxicological responses resulting from exposure to inorganic As. These include type 2 diabetes^34^, a mortality follow-up of a population with baseline urine measurements which found a significant association with lung cancer^35^ and increased genotoxicity measured by micronuclei frequency in urothelial cells^36^. Urinary As biomonitoring, albeit on a small number of volunteers and in relation to soil and dust exposure, has been carried out in Cornwall on two previous occasions^37^,^38^ and elevated concentrations were observed relative to control areas with low environmental As.

A number of considerations need to be taken into account when urinary As is used as a biomarker of exposure. Firstly, total urinary As results can be influenced by high concentrations of arsenobetaine (AB), an organo-arsenical found in seafood, widely thought to be non-toxic^39^ and readily excreted unaltered following dietary intake. This makes it necessary to perform speciation analysis on urine samples to quantify the individual As species and exclude the contribution from AB which does not reflect exposure to more hazardous environmental inorganic As. Secondly, the variation in hydration status among volunteers means that both first morning void (FMV) and spot urine samples differ markedly in their dilution, both giving imperfect estimates of 24 hr excretion^40^. Therefore, in order to be used as a robust indicator of exposure, urinary As concentrations require adjustment for dilution to eliminate variation from fluid balance. Creatinine and specific gravity (SG) adjustment are widely used, but both methods are susceptible to interferences. Variation in urinary creatinine has been demonstrated between demographic groups^41^ and in response to variations in muscle mass^42^ and malnutrition^43^, while possibly a more relevant deterrent of applying this adjustment factor is its observed relationship with As methylation efficiency^44^. Alternatively, because SG is routinely measured by refractometry, the presence of urinary solutes such as protein (proteinuria), glucose (glucosuria) and ketones (ketonuria) alters the refractive index of the liquid irrespective of its dilution, thus giving inaccurate dilution estimates^45^. One alternative adjustment factor, seldom used in biomonitoring studies, is urinary osmolality. Previously overlooked due to the lack of widespread availability and relative cost of the instrumentation required^46^, osmolality is regarded as the ‘gold standard’ and definitive measure of urinary concentration in the clinical and veterinary sciences community^47^. In the case of cryoscopic osmometry, freezing point depression is measured. Freezing point is a colligative property reflective of solute content, expressed here by osmolality (osmoles of solute per mass unit of solvent) and is not vulnerable to the same interferences as SG measurement by refractometry. Given the absence of 24 hr or timed excretion data in the present study, osmolality adjustment was preferred over the two alternative options.

This study aimed to: (1) assess human exposure to inorganic environmental As in a population of PWS users in Cornwall using non-invasive urinary As exposure biomonitoring, (2) assess to what extent the biomarker response can be attributed to PWS drinking water as an exposure route and (3) observe the effect of osmolality adjustment to better define the relationship between urinary As and PWS drinking water As.

## Results

### Study group demographics

**Figure 1:**
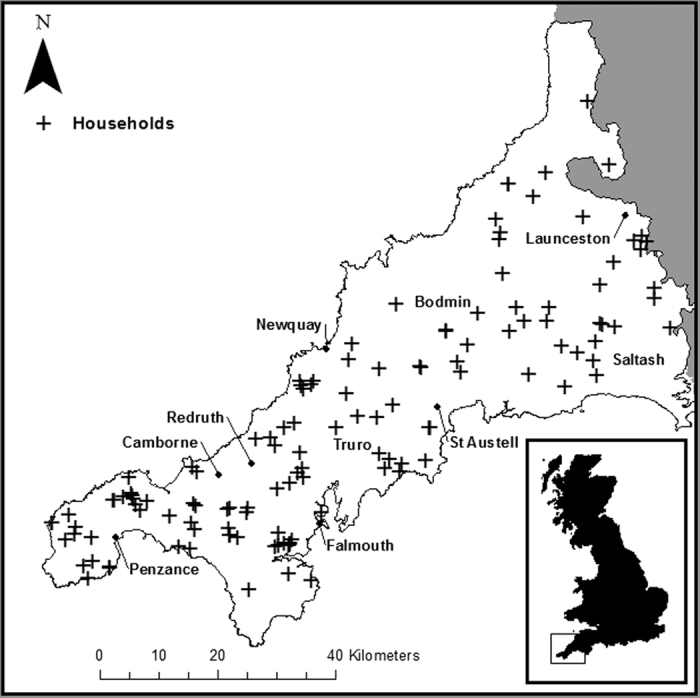
Spatial distribution of sampled households. Compiled using ArcMap 10.1.

**Figure 2:**
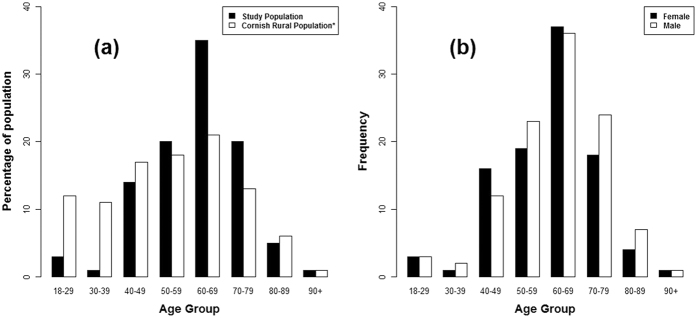
Study group age and gender distribution. While population risk assessment is not the focus of this aspect of the study, it is noted for future reference that the present sample is not wholly representative of the underlying population of rural Cornwall. *Office for National Statistics (ONS) Rural-urban classification 2011 (RUC11) was used to determine the underlying population (see supplementary information). Adapted from data from the Office for National Statistics licensed under the Open Government Licence v.3.0.

The extent of the study area and spatial distribution of households is shown in Fig. 1. A total of 215 volunteers from 129 households participated in the study. Of these volunteers, 207 from 127 households consisting of 108 males (52%) and 99 females (48%), reported using their water for drinking and provided both a drinking water and urine sample. Henceforth, unless otherwise stated, this sub-group will be the focus of the present article. The mean volunteer age was 62 years old (range: 18–90). The age and gender distribution is shown in Fig. 2. The study group was classified as a 99% rural population (see supplementary information).

### PWS drinking water and urine samples

**Figure 3:**
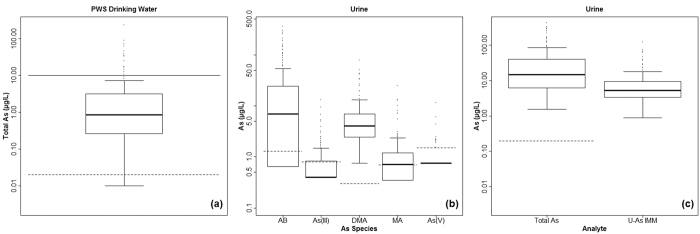
Box and whisker plots of private water supply (PWS) drinking water and urinary As. (**a**) Total As in drinking water samples plotted with its analytical limit of detection (LOD) (lower dashed line) and the UK As PCV (upper solid line). (**b**) Individual urinary As species plotted with their respective LODs (dashed lines). (**c**) Urinary total As and urinary sum of species excluding AB (U-As^IMM^). Boxes range from 1^st^ to 3^rd^ quartiles with a median line, lower and upper whiskers are the lowest and highest datum within 1.5 inter quartile range (IQR) of the lowest and upper quartile respectively and circles are outliers. For plotting purposes, speciation data were censored by replacing <LOD values with ½ LOD.

Summary statistics for total As in drinking water samples and total and speciated As in urine samples (unadjusted and osmolality adjusted) are displayed in Table 1 and plotted in Fig. 3. Geometric means (GM) were calculated in addition to arithmetic means (AM) as the data were positively skewed. Of the 127 households, 126 (99%) had detectable (>0.02 μg/L) As in their drinking water, 62 (49%) had ≥1 μg/L and 15 (12%) exceeded the current WHO guidance value^48^ and UK PCV^23^ of 10 μg/L. This corresponds to 21 of the 207 (10%) volunteers being exposed to drinking water As concentrations above 10 μg/L. The maximum PWS drinking water arsenic concentration was 233 μg/L.

All volunteers had detectable (>0.2 μg/L) concentrations of unadjusted urinary total As; with a maximum observed concentration of 426 μg/L. Speciation data yielded a 98% mean recovery of total As and precision, expressed as relative standard deviation (RSD), was 9%. Despite requesting volunteers to refrain from eating seafood for the 4 days prior to sample collection, a large contribution of total As was from organic AB. Arsenobetaine was detected (LOD 1.3 μg/L) in 152 (73%) samples whilst the mean contribution of AB to total urinary As was 49%; (range: 0–98%). Findings of inorganic As^III^ and As^V^ were lower, with 56 (27%) and 10 (5%) of samples having detectable concentrations (>0.8 μg/L; >1.5 μg/L) respectively. The sum of As^III^ and As^V^ ranged from <LOD (0.8 μg/L and 1.5 μg/L respectively) to 19.2 μg/L. All samples had detectable concentrations of DMA and 107 (52%) had detectable concentrations of MA. Dimethylarsinate was the dominant arsenic species with the exception of AB. The sum of inorganic As (As^III^ and As^V^) and its organic methylated metabolites (MA and DMA), referred to here as U-As^IMM^, ranged from 0.9 to 124 μg/L with an arithmetic mean (AM) of 9.0 μg/L and a GM of 5.8 μg/L.

Urinary osmolality ranged from 181–1161 mOsm/kg, reflecting a large variation in urinary dilution amongst volunteers. Post osmolality adjustment, AM urinary total As moderately decreased from 36.8 to 36.1 μg/L and the GM slightly increased from 15.8 to 17.1 (range: 2.2–404 μg/L). The osmolality adjusted U-As^IMM^ AM and GM also decreased to 8.6 and increased to 6.3, respectively.

Additionally, 30 (14%) urine samples were collected as spot samples at the time of visit as opposed to first morning voids (FMV). To address this, a Welch’s independent two-group t-test was used to assess the difference between the two collection methods. For unadjusted and osmolality adjusted U-As^IMM^ and urinary osmolality no significant difference was observed (*P* = 0.20, *P* = 0.30 and *p* = 0.43, respectively).

### Correlation analysis

**Figure 4:**
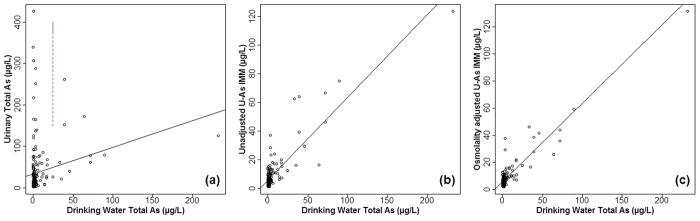
Unadjusted and adjusted urinary As – PWS drinking water As. (**a**) Unadjusted urinary total As. (**b**) Unadjusted U-As^IMM^ (adjusted for AB). (**c**) Osmolality adjusted U-As^IMM^. Linear regression lines are for reference only. A poor relationship between drinking water total As and unadjusted urinary total As is evident (**a**) due to seafood intake and the large contribution of AB on urinary total As results. This is illustrated by the red dashed line showing high urinary total As results at low drinking water As exposure.

**Figure 5:**
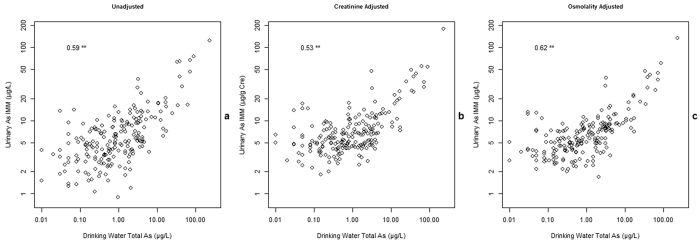
Comparison of alternative U-As^IMM^ adjustment methods. The comparison between unadjusted results (**a**), creatinine adjusted results (**b**) and osmolality adjusted results (**c**). The Spearman correlation is stronger in osmolality adjusted results than both alternatives.

**Figure 6:**
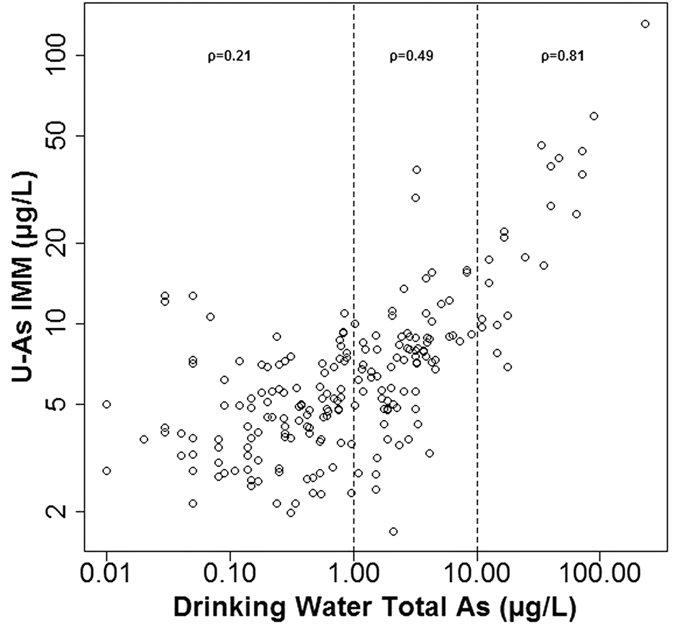
Log-log plot of U-As^IMM^ vs PWS drinking water As divided into drinking water exposure levels. Variables from Fig. 3c plotted on log scale axes to show contrasting exposure-response relationships of participants exposed to different concentrations of As in drinking water. Spearman’s correlation coefficients (ρ) are displayed for the different drinking water As ranges.

Scatterplots showing urinary As vs PWS drinking water total As, both before and after AB and dilution adjustment, are shown in Fig. 4. Figure 4a shows that total As in drinking water was not a good predictor of urinary total As, with a large variation in urinary total As even for volunteers with low PWS drinking water As concentrations. However, when corrected for AB (Fig. 4b) a more positive correlation was observed. Correcting for urinary dilution using osmolality measurements further improved the correlation between urinary As (U-As^IMM^) and PWS drinking water As (Fig. 4c). To test the strength of these correlations, Spearman’s rank correlation coefficient was used as both variables were non-normally distributed (Shapiro-Wilk test: *P* < 0.001 for drinking water total As, and both unadjusted and osmolality adjusted urinary total and U-As^IMM^) and the results from this analysis are shown in Table 2. Following adjustment for AB, a stronger correlation was observed between drinking water and urine samples (Spearman’s ρ = 0.36 (*P* < 0.001) and 0.58 (*P* < 0.001) pre and post AB exclusion respectively). This correlation strengthened slightly (Spearman’s ρ = 0.62, *P* < 0.001) following osmolality adjustment. The correlation between creatinine adjusted U-As^IMM^ (μg/g Cre) and drinking water As is also shown in comparison to unadjusted and osmolality adjusted results (Fig. 5) and is weaker (Spearman’s ρ = 0.53, *P* < 0.001) than both. In addition, correlations were calculated on subsets of different drinking water As concentrations and were found to weaken with decreasing concentration. For drinking water As versus osmolality corrected U-As^IMM^, Spearman’s ρ was 0.81 (*P* < 0.001) when drinking water As was >10 μg/L compared to 0.21 (*P* = 0.031) when <1μg/L. This is shown in Fig. 6.

Finally, 74 households consisted of >1 volunteer, all of whom were included in correlation analyses. Volunteers (observations) sharing a household (sampling unit) were therefore not independent. Correlations between U-As^IMM^ concentrations of volunteers from the same household (*n = *74) were calculated as ρ = 0.59 (*P* < 0.001) and ρ = 0.66 (*P* < 0.001) for unadjusted and osmolality adjusted concentrations, respectively. This had the potential to influence the strength of correlations and, therefore, correlations were re-calculated by randomly selecting one volunteer per household for inclusion. These results are presented in Table 3 and, although some correlations (particularly those calculated for lower drinking water As concentration groups) were numerically different, the overall pattern remained the same. Furthermore, the correlations re-calculated on osmolality adjusted U-As^IMM^ concentrations agreed strongly across drinking water concentrations groups with those originally calculated with the inclusion of all volunteers.

## Discussion

The present study shows that exposure to inorganic As in drinking water, although not widespread, is occurring within the Cornwall study population with 10% of the present study group exposed to >10 μg As/L in drinking water. Although not a true representation of the actual proportion of population exposure, this study builds on the findings^22^ of its precursor survey by confirming human exposure from PWS that exceeded the PCV, with high As concentrations in drinking water reflected by dilution and AB adjusted U-As^IMM^.

The maximum U-As^IMM^ concentration measured in the present study (124 μg/L) was comparable with values found in West Bengal^49^, one of the world’s worst affected regions, some of the highest recorded elsewhere in Europe^50^, and was higher than any found previously in Cornwall^37^,^51^. In 1998 Kavanagh and co-workers^51^ reported a range of 2.7–58.9 μg As/g creatinine (U-As^IMM^) in urine collected from residents (8 boys aged 3–8; 9 adults aged 30–43) of Gunnislake, Cornwall, although the drinking water supply status of the volunteers was not reported. This demonstrates that the larger sample population in this study revealed further exposure incidences in the region and a previously uninvestigated exposure route, both in Cornwall and the UK to date.

Correlation analysis of exposure and response variables showed that the strength of the correlation between drinking water and U-As^IMM^ reduced with decreasing levels of exposure to total As in drinking water. Variation among U-As^IMM^ results in volunteers with <1 μg/L in drinking water was evident, with some urinary U-As^IMM^ results still higher than 10 μg/L. As mentioned, Cornwall is an area of high environmental As and these observations suggest that in low drinking water As concentration scenarios, confounding exposure variables such as direct soil ingestion from home grown produce consumption, dust ingestion/inhalation or contact with high As bearing mine wastes could be more prominent. The importance of these exposure routes will be the focus of further research incorporating the analyses of garden/vegetable patch soils and household dust.

Additionally, with the exception of AB, DMA was the dominant species measured in urine samples. This is not unexpected, as DMA is the major endpoint of As metabolism in mammals, typically accounting for 60–80% of stable urinary As species excluding AB^52^. This outcome requires further consideration given the low drinking water concentrations of the majority of individuals. This is in agreement with the study of Leese *et al*.^29^ who reported high concentrations of AB in urine samples from an unexposed population^29^, in which DMA was also the dominant species after AB. Given the unexposed status of their study population, Leese *et al*.^29^ conclude that dietary sources are responsible for the presence of DMA as well as AB. In addition, they advise that organic methylated species in urine samples do not necessarily indicate exposure to inorganic As. In the case of individuals not exposed to As in their drinking water, future efforts should be made to model the proportion of DMA likely to derive from direct dietary intake versus that excreted as a product of the metabolism of inorganic species.

No robust reference value for U-As^IMM^ applicable to a UK population currently exists and existing values applicable elsewhere are discussed. Commonly cited is the Agency for Toxic Disease Registry (ATSDR) 100 μg/L total urinary As^53^. This was not selected for comparison in the present study due to the large contribution of AB to urinary total As and unless seafood consumption can be categorically ruled out then this value is not recommended. Of 207 urine samples, 12 (6%) exceeded 21.5 μg/L of unadjusted U-As^IMM^, the approximate creatinine adjusted concentration found in a recent study^35^ to correspond to a lung cancer hazard ratio (HR) of 2.0 which is equivalent to double the risk of developing the disease. This value is more appropriate for comparison as it is not affected by AB, however it is noted that because it refers to creatinine adjusted urinary As results from a sample of almost 4000 American Indians, it is not directly applicable to the group studied here. An arguably more appropriate value is the occupational biological effect index (BEI) provided by the American Conference of Government Industrial Hygienists^54^ (ACGIH) (35 μg/L of unadjusted U-As^IMM^), of which 8 (4%) samples exceeded. Whilst acknowledging that this was derived for use with occupational exposure, the BEI was chosen as the comparison value provided to volunteers on feeding back their individual urinary As results (unadjusted U-As^IMM^).

In order to assess the magnitude of exposure it is important to consider how the sample in the present study relates to the underlying population of PWS users in Cornwall and elsewhere in the UK. As demonstrated in Fig. 2a, the sample of volunteers obtained in the present study was biased and is unlikely to reflect the true proportion of exposure in the underlying population. Furthermore, high-As bearing PWS were over-sampled to ensure that a range of exposure scenarios were captured to model the biomarker response. Therefore, the proportion of drinking water As PCV exceedances in the present study is higher than that observed in the wider population of PWS users (12% in the present biomonitoring study versus 5% in the 2011–2013 PWS survey). The relationship between the current sample and the underlying population is a matter for further investigation.

Urinary As biomonitoring is useful in assessing recent exposure^25^, and therefore results offer a snapshot of a relatively narrow exposure window, especially given that FMV/spot samples were taken as opposed to 24 hr collections, making it impossible to assess day-to-day variation in individual excretion patterns. Chronic exposure to As cannot be fully assessed by exposure incidence alone, an assessment of longevity is also needed. The analysis of alternative biomarkers such as hair and toenails is ongoing and may provide evidence of longer term exposure, as will analysis of the temporal stability of As in drinking water samples.

In conclusion, it has been demonstrated that, following the necessary adjustments of urinary As concentrations for AB intake and urinary fluid balance, a strong positive correlation was observed between As concentrations in PWS drinking water and urinary As excretion-indicative of ongoing human exposure to inorganic As in PWS drinking water in Cornwall. Given the comparisons to existing guidance values for other populations, the results of the present study are a cause for concern, albeit for a minority of cases. Efforts should be made to raise wider public awareness of the potential hazards associated with PWS usage and, where analytes exceed the PCV, recommendations for treatment should be made given that it has been demonstrated^55^ that installation of appropriate treatment systems is effective in reducing exposure to As and other elements. This work has raised points for further investigation which should include: whether chronic/long-term exposure is evident; the importance of additional exposure routes; further refinement of As biomonitoring techniques to account for dietary sources of organic As species in addition to AB; identification of specific population groups at risk. Such groups may be dictated geographically or as a result of individual susceptibility or behavioural risk factors. Particular ‘hotspots’ of high exposure require identification using spatial/geostatistical methods and ongoing questionnaire analysis. Finally, the health implications of PWS usage in the UK warrant more investigation by detailed analysis of supply distribution, consumption patterns, geochemical risk modelling in conjunction with health surveillance datasets.

## Methods

### Ethical approval and consent

In accordance with approved guidelines, written informed consent was obtained from all volunteers and only those who were able to provide such were included in the study. In addition, all methods were followed in accordance with approved guidelines. Ethical approval for the study was provided by the University of Manchester Research Ethics Committee (Ref 13068) and the NHS Health Research Authority National Research Ethics Committee (NRES) (Ref 13/EE/0234).

### Sampling strategy and recruitment methods

The sampling frame consisted of volunteers previously involved in the 2011–2013 PWS survey carried out by the BGS on behalf of the former Health Protection Agency (HPA), now part of Public Health England (PHE). Households with a PWS at which volunteers resided formed the sampling units. Observational units consisted of those individual volunteers who met the following inclusion criteria: ≥18 years of age; did not suffer from a health condition that could prevent them from participating in the study; had not been identified from the previous phase as unwilling/unable to participate further; provided informed consent. Prospective volunteers were contacted via an information/invitation letter prior to receiving a telephone call. All of those with >1 As μg/L being found in their drinking water in the previous survey were contacted to include as many as possible in the study. Numbers were then made up with households in the <1 As μg/L category. This approach was designed to maximise the range of observed exposures in the study group.

### Sample collection and pre-treatment

Household visits were made to volunteers by sampling teams. Urine and point of use drinking water samples were collected and an exposure assessment questionnaire administered to volunteers using Microsoft Access 2007 on a laptop/tablet device to ascertain whether volunteers were using their PWS for drinking.

Drinking water samples were collected by running the tap most frequently used for drinking for a minimum of 3 minutes to purge any standing water from the pipes before collecting the water in pre-rinsed (with the water being sampled) LDPE containers (Nalgene, USA). Samples were stored in a cool box during transit. Samples were acidified with 1% v/v HNO_3_ on return to the field laboratory, and then with an additional 0.5% v/v of HCl on return to the Inorganic Geochemistry Facility at the British Geological Survey.

For urine collection, volunteers were asked to refrain from eating seafood for a minimum of 4 days prior to providing a sample. HDPE containers (60 mL) (Nalgene, USA) were mailed in advance to volunteers who were asked to provide a FMV, mid-stream urine sample on the day of their visit and store it in the refrigerator until collection by the sampling team. Where instructions were not followed (*n* = 30), a spot urine sample was collected at the time of the visit where possible. Samples were stored in a cool box during transit and, on return to the field laboratory, filtered through 0.45 μm Acrodisc^®^ syringe filters (PALL Life Sciences, USA) into 30 mL HDPE containers (Nalgene, USA) and then frozen at −30 °C until analysis.

### Reagents and standards

The aqueous solutions used throughout the study were prepared using 18.2 MΩ deionised water (Millipore, UK). Nitric (HNO_3_) and hydrochloric (HCl) acids were Romil-SpA™ super purity grade (Romil, UK). Ammonium nitrate (NH_4_NO_3_) solutions were prepared from a solid stock of BioXtra ≥99.5% purity (Sigma-Aldrich, USA) and pH adjusted using Aristar^®^ grade 25% ammonia (NH_3_) solution (BDH, UK). Arsenic calibration standards were prepared from an in-house multi-element stock in which the As contribution was from a 1000 mg/L PrimAg^®^ grade mono-elemental stock solution (Romil, UK). Arsenic QC standards (5 μg/L) were prepared from a multi-element stock solution of various concentrations with As at 20 mg/L (Ultra Scientific, USA). A Tellurium (Te) ICP-MS internal standard was prepared from a PlasmaCAL 10,000 mg/L stock solution (SCP Science, Canada). The following standards were used for the calibration of individual As species as follows: As^III^: 1000 As mg/L stock solution of arsenic trioxide (As_2_O_3_) (Inorganic Ventures, USA); As^v^: 1000 As mg/L stock solution of arsenic (V) oxide hydrate (As_2_O_5_·xH_2_O) (Inorganic Ventures, USA); MA: 50 As mg/L in-house stock solution of monomethylarsonic acid ((CH_3_AsO(OH)_2_) prepared from solid (Sigma-Aldrich, USA); DMA: 50 As mg/L in-house stock solution of dimethylarsinic acid ((CH_3_)_2_AsO(OH)) prepared from solid (Greyhound Chromatography, UK); AB: 1031 As mg/L BCR-626 standard solution of arsenobetaine ((CH_3_)_3_As^+^ CH_2_COO^−^) (LGC, UK).

### Total arsenic determination by ICP-MS

Urine samples were thawed at room temperature and refrigerated at 4 °C prior to analysis. Due to the high matrix of urine, samples (1 mL) were diluted x10 with 1% v/v HNO_3_ and 0.5% v/v HCl to reduce the effects of high concentrations of sodium (Na) on signal stability. Acidified PWS drinking water samples were refrigerated at 4 °C prior to analysis and analysed neat. Total As concentrations in both water and urine samples were determined using inductively coupled plasma mass spectrometry (ICP-MS). An Agilent 7500 Series ICP-MS instrument (Agilent Technologies, USA) was used under the operating conditions described by Watts *et al*.^56^. The instrument was fitted with a MicroMist low-flow nebulizer (Glass Expansion, Australia) and sample introduction was accelerated using an ASXpress rapid sample introduction system (Teledyne CETAC Technologies, USA). A three-point calibration was used with As concentrations at 1, 10 and 100 μg/L. Arsenic was detected in helium (He) collision cell mode to reduce potential mass 75 polyatomic interferences such as argon chloride (^40^Ar^35^Cl^+^). A Te internal standard was introduced simultaneously via a T-piece and the Te signal response used to fit urinary As data. The limits of detection (LOD) were calculated as 3σ of analytical run blanks and were 0.02 and 0.2 As μg/L for drinking water and urine samples respectively.

### Arsenic speciation by HPLC-ICP-MS

**Figure 7:**
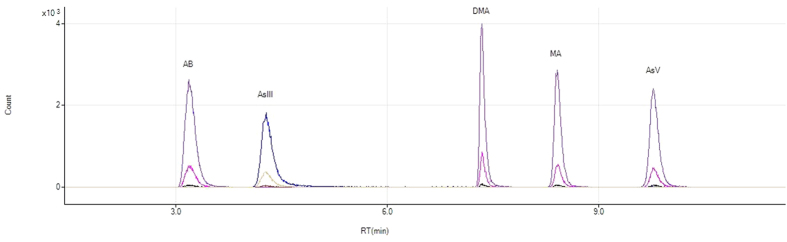
Arsenic speciation standard chromatogram. Chromatograms obtained for standard calibration solutions at 1, 10 and 50 μg/ L. Calibration of arsenate (As^V^), methylarsonate (MA), dimethylarsinate (DMA) and arsenobetaine (AB) was performed with mixed solutions of arsenic (V) oxide hydrate (As_2_O_5_·xH_2_O), monomethylarsonic acid ((CH_3_AsO(OH)_2_), dimethylarsinic acid ((CH_3_)_2_AsO(OH)) and arsenobetaine ((CH_3_)_3_As + CH_2_COO-) respectively. Calibration of arsenite (As^III^) has been plotted simultaneously and was achieved with separate solutions of arsenic trioxide (As_2_O_3_).

Urine samples (150 μL) were diluted × 10 with deionised water and As speciation was measured using high performance liquid chromatography coupled to ICP-MS (HPLC-ICP-MS) using the method described by Button *et al*.^57^. In summary, a GP50 gradient pump and an AS auto-sampler (Dionex, USA) were coupled to the ICP-MS instrument with PEEK tubing. Chromatography was performed with a PRP-X100 anion exchange column and a PRP-X100 guard column (Hamilton, USA) using gradient elution with the mobile phase (pH 8.65, 1 mL/min) alternating between 4 and 60 mM NH_4_NO_3_. A 3-point calibration was used with 1, 10 and 50 As μg/L solutions of As^III^ and a mixed solution of 1, 10 and 50 As μg/L As^V^, MA, DMA and AB. Figure 7 shows a standard chromatogram obtained for calibration solutions. The LODs for this method (3σ of blank values) are reported by Watts *et al*.^58^: 0.8; 1.5; 0.7; 0.3; 1.3 As μg/L for As^III^, As^V^, MA, DMA and AB respectively. It is noted that this method cannot distinguish the trivalent and pentavalent forms of both MA and DMA which vary in genotoxicity^59^.

### Urinary dilution measurement and adjustment factor

Urinary osmolality was measured using an Osmomat 030 cryoscopic osmometer (Gonotec, Germany). The osmolality of the urine samples was determined by comparative measurement of their freezing point with that of pure water. The normalisation procedure applied was adapted from that used for creatinine and SG in a recent study on the normalization of urinary drug concentrations^60^, and based on the Levine-Fahy equation^61^ as follows:


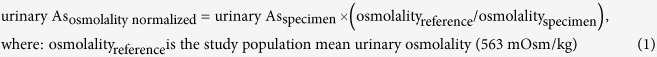


### Quality control (QC)

Field duplicates for drinking water (5% of samples) and urine (4% of samples) were collected with the following mean percentage differences (where analytes >LOD): drinking water total As: 7% (*n* = 6), urinary total As: 10% (*n* = 9), AB: 6% (*n* = 6) and MA: 13% (*n* = 2). Inter-run duplicates were analysed for urinary total As and AB to assess method reproducibility (total As mean percentage difference: 3% (*n* = 6), AB: 8% (*n* = 12). To assess signal stability and the possibility of drift resulting from high urinary matrices, intra-run duplicates were analysed for urinary total As and speciation (species >LOD) (total As mean percentage difference: 8% (*n* = 5), AB: 2% (*n* = 5), MA: 2% (*n* = 1), DMA: 5% (*n* = 6). Certified reference materials (CRM) were analysed with drinking water and urine samples: NIST SRM 1643e Trace Elements in Water (National Institute of Standards and Technology, USA) (certified value: 58.98 ± 0.70 As μg/L, recovery: 100% (*n* = 4), precision: 3%) and NIES No.18 Human Urine (National Institute for Environmental Studies, Japan) (total As certified value: 137 ± 11 As μg/L, recovery: 99% (*n* = 14), precision: 5%, AB certified value: 69 ± 12 As μg/L, recovery: 92% (*n* = 18), precision: 5%, DMA certified value: 36 ± 9 As μg/L, recovery: 115% (*n* = 18), precision: 12%). Independent matrix matched QC standards (total As: 5 μg/L) were also analysed with urine samples (recovery: 94% (*n* = 16), precision: 8%). Background contamination was monitored using run blanks for urine and drinking water analyses, reagent (acid) blanks for drinking water analysis and filter blanks for urine analysis. Duplicate measurements were made on 12% (*n* = 25) of urine samples for osmolality with a mean percentage difference of 1%.

### Statistical analysis

Statistical analysis (including the production of exploratory plots) was performed using R version 3.0.0 (base package)^62^. Welch’s independent two sample t-test was used to assess the difference between results of spot and FMV urine collections. A Shapiro-Wilk test was used to determine the normality of exposure and outcome variables before and after applying log transformation. Correlation tests were performed using Spearman’s rank correlation coefficient accompanied by a significance test to exclude the possibility of the observed correlations resulting from random sampling. Descriptive statistics, with the exception of the geometric mean, were obtained using the ‘psych’ package^63^. In the case of speciation data, where manual peak integration resulted in samples with zero or negative values for particular species (As^III^ and As^V^), left censoring was required to enable data for log transformation and the calculation of geometric means. Values <LOD were therefore replaced with that of half the appropriate LOD.

### Mapping

All maps displayed as figures in this manuscript were compiled using ESRI ArcGIS Desktop version 10.1. (ArcMap) Environmental Systems Research Institute. Redlands, CA.

### Dissemination of results to households

A letter containing individual result data was fed back to households. Where a PCV exceedance was highlighted, specific advice was provided to participants on any potential health risks and suggested corrective actions were given. All participants were provided with appropriate contact details for any follow-up enquiries.

The letter and guidance were developed by PHE along with BGS and the Local Authority. The letter was sent from the Local Authority, as the regulator for PWS in England.

## Additional Information

**How to cite this article**: Middleton, D. R. S. *et al*. Urinary arsenic profiles reveal substantial exposures to inorganic arsenic from private drinking water supplies in Cornwall, UK. *Sci. Rep*. **6**, 25656; doi: 10.1038/srep25656 (2016).

## Supplementary Material

Supplementary Information

## Figures and Tables

**Table 1:** Descriptive statistics for drinking water and urinary arsenic concentrations.

*n*	Drinking water total As (μg/L)	Urinary total As (μg/L)	Urinary U-AsIMM (μg/L)	Urinary AB (μg/L)	Urinary AsIII (μg/L)	Urinary AsV (μg/L)	Urinary MA (μg/L)	Urinary DMA (μg/L)
	127	207	207	207	207	207	207	207
Unadjusted								
Arithmetic mean	7.0	37	9.0	26	0.7	0.5	1.3	6.5
Geometric mean	1.0	16	5.8	6.2	0.6	0.8	0.7	4.3
Median	0.9	15	5.3	6.9	0.3	0.3	0.7	4.0
Min	<LOD	1.6	0.9	<LOD	<LOD	<LOD	<LOD	0.8
Max	233	426	124	363	13	12	25	79
Osmolality Adjusted								
Arithmetic mean		36	8.6	26	0.6	0.5	1.2	6.3
Geometric mean		17	6.3	6.5	0.6	0.8	0.7	4.7
Median		15	5.7	7.3	0.4	0.3	0.8	4.4
Min		2.2	1.7	<LOD	<LOD	<LOD	<LOD	1.4
Max		404	131	360	14	9.1	27	84

Drinking water As results are shown on the basis of collected samples/sampled supplies (*n* = 127). Between 1 and 4 volunteers were associated with any one drinking water sample. Urinary As results are shown both with and without osmolality adjustment.

**Table 2:** Correlation analysis of exposure and outcome variables for all volunteers.

	Spearman’s ρ (*P* value)			
	Drinking Water As < 1 μg/L (*n* = 109)	Drinking Water As1–10 μg/L (*n* = 77)	Drinking Water As > 10 μg/L (*n* = 21)	Full range(*n* = 207)
Drinking Water As vs Urinary Total As	0.19 (*P* = 0.048)	0.36 (*P* = 0.001)	0.55 (*P* = 0.009)	0.36 (*P* < 0.001)
Drinking Water As vs U-As^IMM^	0.18 (*P* = 0.060)	0.38 (*P* < 0.001)	0.69 (*P* < 0.001)	0.58 (*P* < 0.001)
Drinking Water As vs Osmolality Adjusted U-As^IMM^	0.21 (*P* = 0.031)	0.49 (*P* < 0.001)	**0.81** (*P* < 0.001)	0.62 (*P* < 0.001)

A strong correlation (bold font) is only observed for U-As^IMM^ (osmolality adjusted) for drinking water As >10 μg/L. All household volunteers were included in analyses.

**Table 3:** Correlation analysis of exposure and outcome variables for single volunteers per household.

	Spearman’s ρ (*P* value)			
	Drinking Water As<1 μg/L (*n* = 65)	Drinking Water As1–10 μg/L (*n* = 47)	Drinking Water As>10 μg/L (*n* = 15)	Full range(*n* = 127)
Drinking Water As vs Urinary Total As	0.27 (*P* = 0.03)	0.42 (*P* = 0.004)	0.48 (*P* = 0.07)	0.40 (*P* < 0.001)
Drinking Water As vs U-As^IMM^	0.23 (*P* = 0.06)	0.49 (*P* < 0.001)	0.69 (*P* = 0.005)	0.60 (*P* < 0.001)
Drinking Water As vs Osmolality Adjusted U-As^IMM^	0.19 (*P* = 0.13)	0.54 (*P* < 0.001)	**0.82** (*P* < 0.001)	0.62 (*P* < 0.001)

A strong correlation (bold font) is only observed for U-As^IMM^ (osmolality adjusted) for drinking water As >10 μg/L. One volunteer per household was chosen at random for inclusion in analyses.
