# Supplementary material for: Urinary arsenic profiles reveal exposures to inorganic arsenic from private drinking water supplies in Cornwall, UK
Source: Sci Rep. 2016 May 9;6:25656. doi: 10.1038/srep25656 (PMC4860641; doi:10.1038/srep25656)
Supplement: Supplementary Information [file srep25656-s1.pdf]

Urinary arsenic profiles reveal substantial exposures to inorganic arsenic from private drinking water supplies in Cornwall, UK

Middleton, D.R.S.[1,2,3], Watts, M.J.[2], Hamilton, E.M.[2], Ander, E.L.[2], Close, R.M.[3], Exley, K.S.[3], Crabbe, H.[3], Leonardi, G.S.[3], Fletcher, T.[3] and Polya, D.A.[1]

Supplementary Information

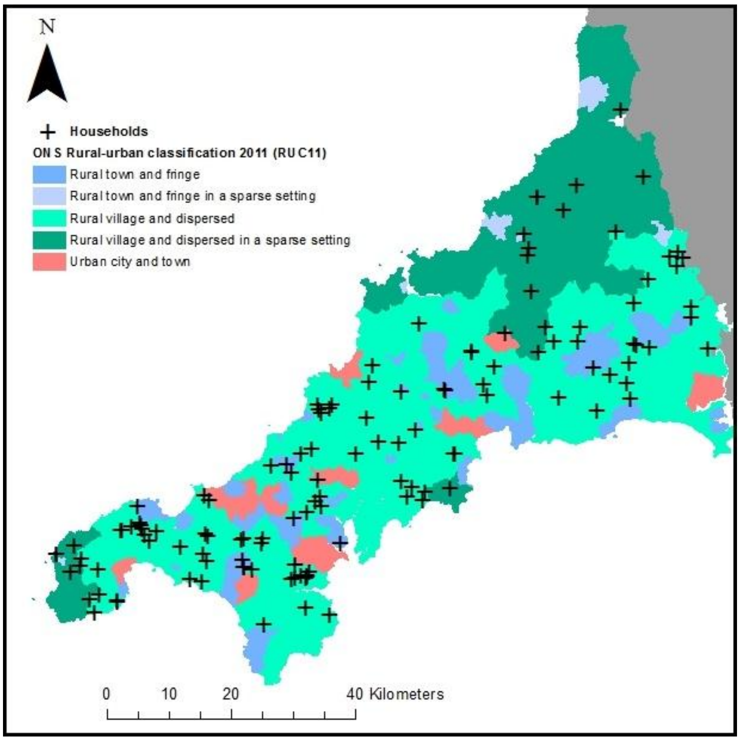

Figure 1 |ONS Rural-urban classification 2011 (RUC11) of study area and households. © Crown copyright and database rights [2015] Ordnance Survey 100019153. Compiled using ArcMap 10.1.

RUC11	Number of households (%)
Urban city and town	3 (2%)
Rural town and fringe	11 (9%)
Rural village and dispersed	96 (76%)
Rural village and dispersed in a sparse setting	17 (13%)

Table 1 | Households categorised by ONS RUC11. As 76% and 13% of households were classified as 'Rural village and dispersed' and 'Rural village and dispersed in a sparse setting' respectively, age-group population statistics for these two categories were used to compare against the study group population.

Source data from the Office for National Statistics licensed under the Open Government Licence v.3.0.
